# Non-Surgically Treated Distal Radius Fractures in the Adult Population: A Systematic Review and Meta-analysis

**DOI:** 10.1177/15589447251415388

**Published:** 2026-02-01

**Authors:** Vili Palola, Rasmus Liukkonen, Matias Vaajala, Ilari Kuitunen, Antti P. Launonen, Ville M. Mattila

**Affiliations:** 1 Tampere University, Finland; 2 Central Finland Central Hospital Nova, Jyvaskyla, Finland; 3 University of Eastern Finland, Kuopio, Finland; 4 Tampere University Hospital, Finland

**Keywords:** distal radius fracture, non-surgical treatment, cast, PROM, PRWE

## Abstract

Distal radius fracture (DRF) is the most common upper extremity fracture, and cast immobilization is the most widely used treatment for DRF. However, prospective outcome data on conservatively treated DRF remain limited. The aim of this systematic review and meta-analysis was to report the pooled patient-reported outcome measures (PROMs) in adult patients with non-surgically treated DRFs. PubMed (Medline), Web of Science, and the Cochrane Central Register of Controlled Trials databases were searched. The primary outcome was to analyze PROMs at 3-month, 6-month, and 12-month follow-ups. The secondary outcomes were to examine the pain, complication rate, and health-related quality of life. A total of 34 studies were included for analysis in this review. Non-surgically treated DRF patients aged ≥18 were included. At 3 months, the pooled mean of the patient-reported wrist evaluation was 31.0. At 12 months, the pooled mean was 10.8. The pooled mean of pain outcome measures was 2.6 at 3 months and 1.8 at 12 months. In conclusion, we found significant improvement in function between the 3-month and 12-month follow-ups in adult patients with non-surgically treated DRF. In addition, 17.9% of patients with non-surgically treated DRFs experience adverse events following treatment.

## Introduction

Distal radius fracture (DRF) is the most common upper extremity fracture.^1^ For the treatment of DRF, the choice between cast immobilization and surgery has been largely debated lately. However, no consensus has been reached regarding the optimal treatment method. Generally, the chosen treatment depends on factors such as the type of DRF, the patient’s age and expectations, activity level, and the patient’s and surgeon’s preferences.

The goal of DRF treatment is to optimize function in an acceptable timeframe while minimizing the risks of complications. Operative treatment is considered to enable an earlier return to normal function.^2^ Therefore, recent studies have mainly focused on physical impairment in grip strength, range of motion, and, more recently, patient-reported outcome measures (PROMs).

Most meta-analyses have concentrated on comparing surgery and nonoperative treatments.^3,4^ Even though DRF is a common injury, our knowledge of recovery after nonoperatively treated DRFs from a prospective perspective is limited. Based on studies comparing conservative treatment to operative treatment, the general consensus suggests that while surgical treatment often results in better anatomical alignment, it does not consistently translate into superior long-term functional outcomes compared with nonoperative management.^3,4^

The aim of this systematic review and meta-analysis was to: (1) report the pooled outcomes with PROMs at various follow-up time points in adult patients with nonoperatively treated DRFs; and (2) report the pooled pain, complication rates, and health-related quality of life (HrQoL) at the specified time points.

## Methods

### Information Sources and Search Strategy

For this systematic review, PubMed (Medline), Web of Science, and the Cochrane Central Register of Controlled Trials (CENTRAL) databases were searched. The literature search was conducted on December 9, 2023, with the terms “(conservative OR nonoperative OR splinting OR casting) AND (distal AND radius AND fracture),” and the search was updated in May 2025 before submission. The review has been reported according to the Preferred Reporting Items for Systematic Reviews and Meta-Analyses (PRISMA) 2020 checklist. The PRISMA checklist can be found as supplementary material (Supplementary File 1). The study protocol was prospectively registered in the PROSPERO database (CRD42023492112). No funding was received for this study.

### Eligibility Criteria and Selection Process

The Patient, Intervention, Control, Outcome measure, and Study design (PICOS) criteria was used for the search of relevant publications. Records from the searched databases were imported into Covidence, and duplicates were automatically removed. The inclusion criteria were randomized controlled trials (RCTs) consisting of patients aged more than 18 years (P) and with nonoperatively treated DRFs (I) in at least one of the study groups. As this meta-analysis was a single-arm meta-analysis, no comparator group was extracted (C). All the studies were included in this review regardless of the type of fracture if they reported outcomes with validated PROMs with at least 1 year of follow-up (O). Furthermore, only RCTs were included (S). This approach was chosen because RCTs provide standardized, prospectively collected data without the selection bias that would be present in a purely nonoperatively treated cohort. All the records from the searched databases were screened, and those abstracts were assessed by 3 authors (VP, MV, IK). After screening the abstracts, we screened full-text articles. Each study was independently assessed by 2 authors, and 3 authors participated in the screening process. In the event of a conflict, the consensus was achieved by the fourth author (RL). Records meeting the inclusion criteria were selected for eligibility assessment. The study was eligible for our analyses if the results were reported with a minimum of 1 year of follow-up.

### Data Extraction

Data were extracted into an Excel spreadsheet (Microsoft Corp., Redmond, WA, USA) by 3 authors (VP, RL, MV). The first 20% of the studies were extracted independently by 2 authors, and after the quality was sufficient, the rest were extracted by 1 author. The extracted data included study characteristics such as title, authors, and year of publication. For each study, we collected data on the included patients’ first and last years, follow-up duration, mean patient age, patient sex, and the number of included injuries. The extracted outcomes included the PROMs, pain, complications, HrQoL, and return to work. These measures were extracted as previously validated questionnaires, such as Disabilities of the Arm, Shoulder, and Hand (DASH) or Short Form 12 (SF-12).

### Effect Measures

The primary outcome of this review was the pooled outcome at 3-month, 6-month, and 12-month follow-ups as measured with the validated PROMs. Only the most commonly used PROMs were included in synthesizing extracted data to produce accurate results. According to the original protocol, the aim was to report the standardized difference to the baseline PROMs, but as no study reported the baseline measurements, this could not be performed. Hence, only the pooled outcomes of such outcomes were reported at the prespecified time points. The secondary aims of this review were to examine the pooled pain, complication rate, and HrQoL with previously specified time points.

### Risk of Bias

Risk of bias (RoB) assessment was performed by 2 authors (RL, MV) using the ROBINS-I (Risk Of Bias In Non-randomized Studies—of Interventions) assessment tool. Although the studies were RCTs, we assessed them primarily in the context of a conservatively treated cohort, which is why we selected ROBINS-I for the evaluation. All the studies were screened independently and conflicts were discussed together. The complete RoB assessment template is provided as a supplementary file 2.

### Statistical Analysis

All included studies were analyzed simultaneously, and pooling was performed using inverse-variance weighting. The variance for each study was calculated as SD^2^/n, assuming a normal distribution of the outcome measure. Statistical heterogeneity was assessed using the *I*^2^ statistics. However, a random-effects model was applied regardless of the *I*^2^ statistics due to the potential variability in treatment protocols across the included studies. Due to the limited number of studies, no subgroup-analyses were performed. Pooled outcomes were reported with 95% confidence intervals (CIs). All analyses were performed using the *meta* package from R v. 4.1.2 (R Foundation for Statistical Computing, Austria).

## Results

**Figure 1.**
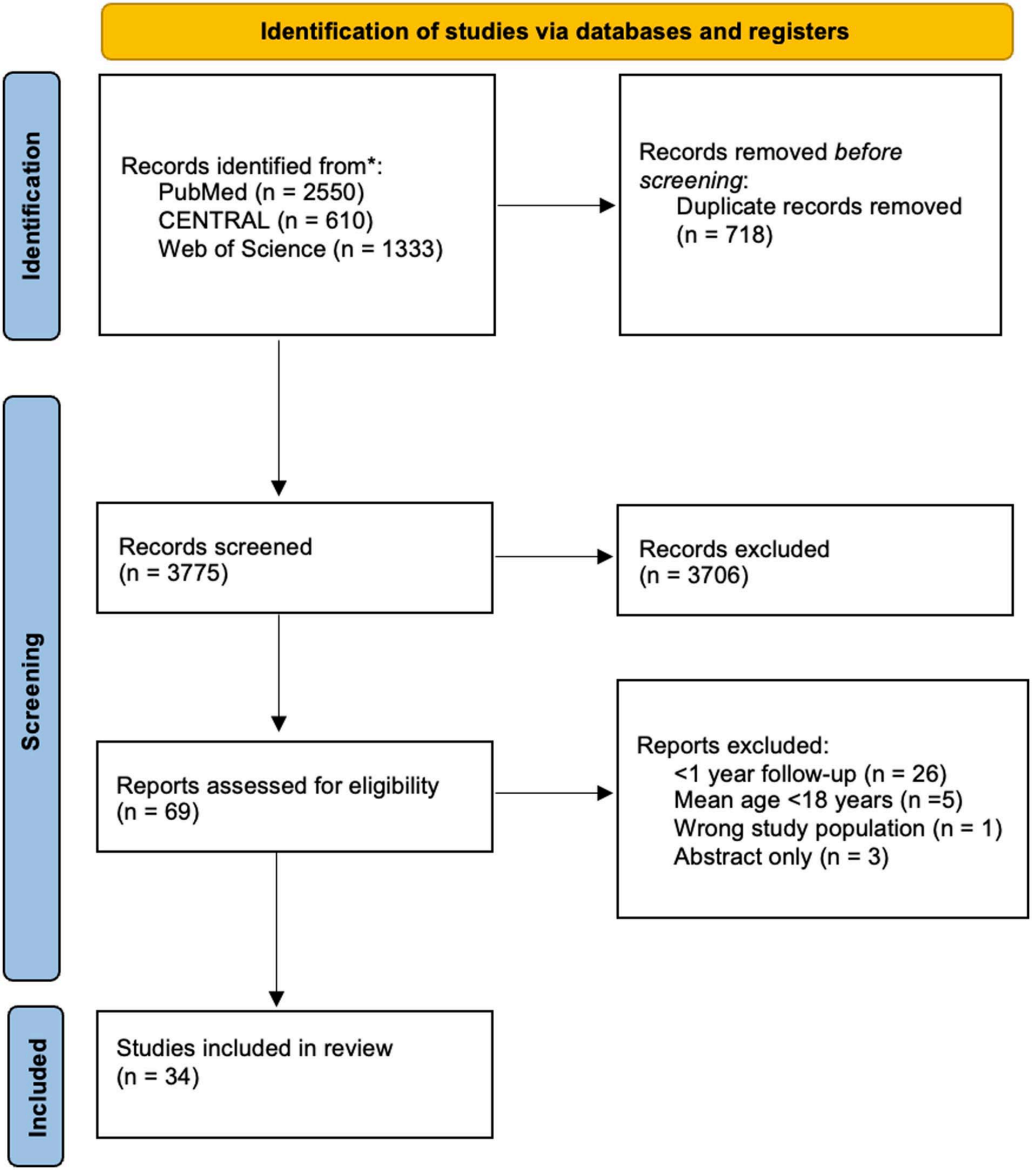
Preferred Reporting Items for Systematic Reviews and Meta-Analyses flowchart of the study selection process.

In total, 4493 studies were eligible for screening. After screening and full-text reading, a total of 34 trials (48 study arms), including 3368 patients with nonoperatively treated DRFs, were included in this review (Figure 1). Of these, a total of 4 studies (6 study arms) were retrieved during the update search.^5-8^ The mean patient age was 65.7 years (range, 18-100 years), and the average duration of cast immobilization was 35 days (range, 7-49 days). The duration of the cast treatment was at least 4 weeks in 67% of the patients. The median number of fractures per study was 45 (interquartile range [IQR] = 34-55), and most of the patients in these studies were women (75.4%, 2471/3278). The earliest included study was published in 2004, whereas the most recent studies were published in 2025. AO classification (standardized system form the AO Foundation for classifying bone fractures) was reported as an inclusion criterion in 67% of studies (22/34). In most studies (33/34), patients presented with displaced fractures requiring reduction. Notably, only 3 out of the 30 studies included non-displaced fractures. Further details of the included studies are presented in Table 1.

**Table 1.** Characteristics of the Included Studies.

Study	Participants, n	Mean age	Women (%)	Extracted outcomes	Follow-up, months	Type of DRF	AO classification
Ahmed et al^5^	17	53.5	13 (77)	PROM, complications	12	Displaced, unstable	A2, A3, C1, or C2
Arora et al^9^	37	77.4	27 (73)	PROM, pain, complications	12	Displaced, unstable	A2-3, C1-3
Ax et al^10^	105	46.1	92 (88)	PROM, pain, complications, HrQoL	24	Colles’, primarily stable	NA
Azzopardi et al^11^	27	71	25 (93)	Pain, HrQoL	12	Dorsally angulated, unstable	A3
Bartl et al^12^	81	74.4	76 (94)	PROM, pain, complications, HrQoL	12	Unstable	C1-3
Barvelink et al^6^	420	62.5	351 (84)	PROM, complications	12	Displaced	NA
Bentohami et al^13^	72	56	49 (68)	PROM, pain, complications	12	Non- or minimally displaced	NA
Boersma et al^14^	40	53.5	29 (73)	PROM, pain, complications, return to work	12	Non- or minimally displaced	NA
Camur et al^15^	140	57.8	86 (61)	PROM, complications	12	Displaced	A1-3, B1-3
Christersson et al^16^	109	65.8	98 (90)	PROM	12	Colles’, dorsally angulated	A3, C2-3
Chung et al^2^	109	76	93 (85)	PROM, pain, complications, HrQoL	12	Displaced	A1-3, C1-3
Chung et al^17^	109	76	93 (85)	PROM, pain, complications, HrQoL	24	Displaced	A1-3, C1-3
Coughlin et al^18^	120	49	80 (67)	Complications	12	3 cm of the radiocarpal joint	NA
De Brujin et al^7^	402	53.5	267 (66)	PROM, pain, complications	12	Non- or minimally displaced	NA
Elbardesy et al^19^	80	61.9	64 (80)	PROM, complications	12	Non- or minimally displaced	NA
Foldhazy and Ahrengart^20^	31	71.5	29 (94)	Complications	12	Displaced, dorsally angulated	A2-3, C1-3
Hassellund et al^21^	50	73.9	42 (84)	Pain, complications, HrQoL	12	Displaced, dorsally angulated	A2-3, C2-3
Haslhover et al^8^	40	77.3	NA	PROM, complications	12	Displaced	C1-2
Hegeman et al^22^	17	69	16 (94)	PROM	12	Unstable, intra-articular	C2-3
Lawson et al^23^	85	71.3	75 (88)	PROM, pain, complications, HrQoL	12	Displaced	A2-3, C2-3
Martinez-Mendez et al^24^	47	70	37 (79)	PROM, pain, complications	24	Displaced, complex intra-articular	C1-3
Mulders et al^25^	43	60	36 (84)	PROM, complications, HrQoL	12	Displaced, extra-articular	A2-3
Olech et al^26^	50	71.2	-	PROM, pain, complications	15	Stable, extra-articular	NA
Raittio et al^27^	105	46.1	92 (88)	PROM, pain, complications, HrQoL	12	Colles’, primarily stable	NA
Reid et al^28^	67	60.1	51 (76)	PROM, HrQoL	12	NA	NA
Saving et al^29^	64	78	56 (88)	PROM, complications, HrQoL	12	Displaced, dorsally angulated	A2-3, C1-3
Selles et al^30^	46	59	40 (87)	PROM, complications, HrQoL	12	Displaced, intra-articular	C1-3
Shaikh et al^31^	529	65	330 (62)	PROM, complications, HrQoL	12	Dorsally displaced	A2-3, C1-3
Sharma et al^32^	32	48.1	18 (58)	PROM, complications	24	Displaced, intra-articular	B, C (without isolated radial styl. fracture)
Sirniö et al^33^	42	64	39 (93)	PROM, complications	24	Displaced	A2-3, C1-2
Tahir et al^34^	72	81	17 (24)	PROM, complications, HrQoL	12	Displaced	A2-3, C1-3
Thorninger et al^35^	50	74	40 (80)	PROM, complications	12	Unstable	NA
Van Delft et al^36^	100	67.6	85 (85)	PROM	12	Displaced	A2-3, C1-3
Wong et al^37^	30	71	25 (83)	PROM, complications, HrQoL	12	Unstable, dorsally angulated, extra-articular	NA

*Note.* DRF = distal radius fracture; PROM = patient-reported outcome measure; HrQoL = health-related quality of life; NA = not applicable.

### Risk of Bias

A total of 5 studies demonstrated having a low risk of bias.^9,12,23,33,36^ None of the included studies exhibited a critical risk of bias in any of the assessed domains. Therefore, all studies were deemed eligible and included in the final analyses. The least sufficient domain in the assessment was bias due to missing data, as 65% (22/34) of the included studies had at least a moderate risk of bias in this domain (Supplementary File 2).

### Patient-Reported Outcome Measures

**Figure 2.**
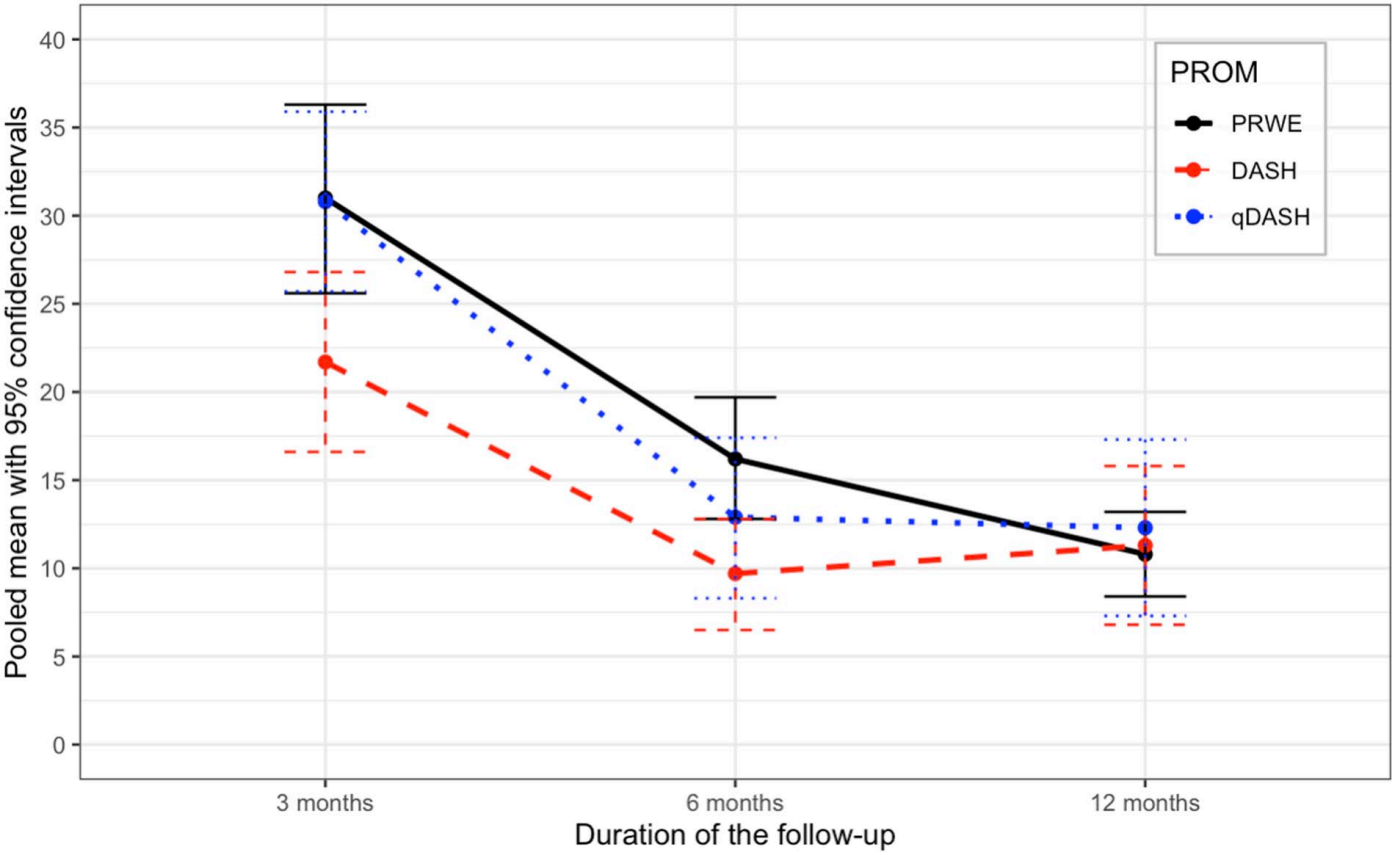
Pooled patient-reported outcome measurements. *Note.* PROM = patient-reported outcome measure; PRWE = patient-reported wrist evaluation; DASH = Disabilities of the Arm, Shoulder, and Hand; qDASH = Quick Disabilities of the Arm, Shoulder, and Hand.

The most used PROM was the patient-reported wrist evaluation (PRWE). At 3 months, 24 study arms reported the outcomes with PRWE. At 6 months, 17 study arms reported the outcomes with the PRWE; at 12 months, 22 study arms reported the outcomes with PRWE. At 3 months, the pooled mean of the PRWE was 31.0 (24 study arms; 2118 patients; CI = 25.6-36.3; *I*^2^ = 99.7%). At 6 months, the pooled mean was 16.2 (17 study arms; 1688 patients; CI = 12.8-19.7; *I*^2^ = 93.8%). At 12 months, the pooled mean was 10.8 (22 study arms; 1963 patients; CI = 8.4-13.2; *I*^2^ = 97.7%). Further details on the pooled outcomes of PROMs are presented in Table 2 (Figure 2).

**Table 2.** Pooled Outcomes as Measured With PROMs Using Random-Effects Model.

Follow-up	Study arms (n)	Patients (n)	Mean (95% CI)	*I* ^2^
**3 months**				
PRWE	24	2118	31.0 (25.6-36.3)	99.7%
DASH	9	356	21.7 (16.6-26.8)	90%
qDASH	5	683	30.8 (25.7-35.9)	95.1%
**6 months**				
PRWE	17	1688	16.2 (12.8-19.7)	93.8%
DASH	6	197	9.7 (6.5-12.8)	68%
qDASH	5	621	12.9 (8.3-17.4)	97.1%
**12 months**				
PRWE	22	1963	10.8 (8.4-13.2)	97.7%
DASH	10	426	11.3 (6.8-15.8)	95.1%
qDASH	7	723	12.3 (7.3-17.3)	98.4%
**>12 months**				
PRWE	3	128	18.8 (7.8-29.3)	89.2%
DASH	1	26	13^a^	—
qDASH	3	128	20.4 (12.6-28.2)	78.1%

*Note.* PROMs = patient-reported outcome measures; CI = confidence interval; PRWE = patient-reported wrist evaluation; DASH = Disabilities of the Arm, Shoulder, and Hand; qDASH = Quick Disabilities of the Arm, Shoulder, and Hand.

a Only one study was included, hence no pooling was possible.

### Pain

At 3 months, the pooled mean of the pain outcome measures was 2.6 (11 study arms; 800 patients; CI = 1.6-6; *I*^2^ = 97.1%). At 12 months, the pooled mean of the pain outcome measures was 1.8 (9 study arms; 793 patients; CI = 0.4-3.2; *I*^2^ = 99.3%).

### Adverse Events

**Figure 3.**
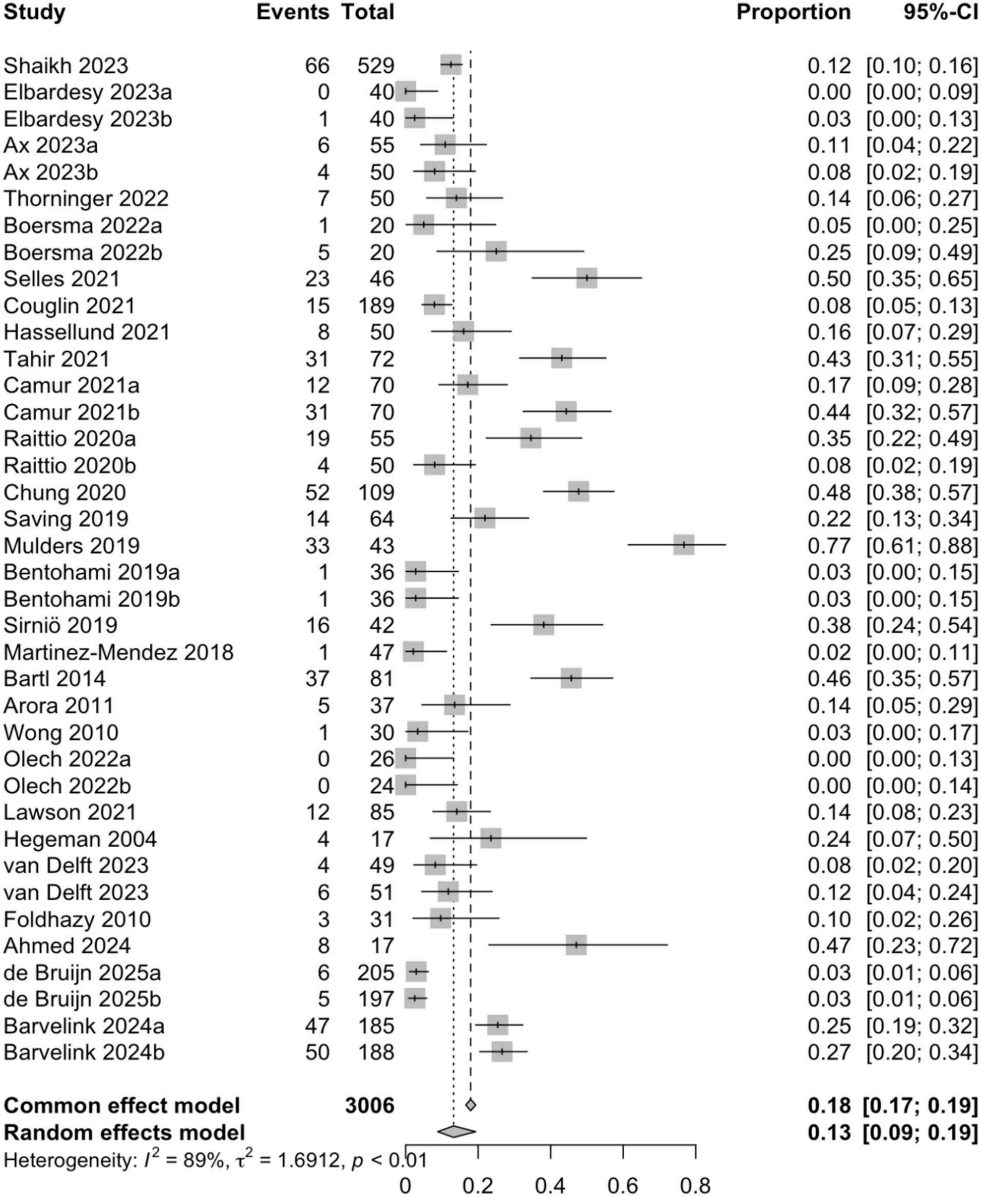
The pooled risk of the adverse events. *Note.* CI = confidence interval.

**Figure 4.**
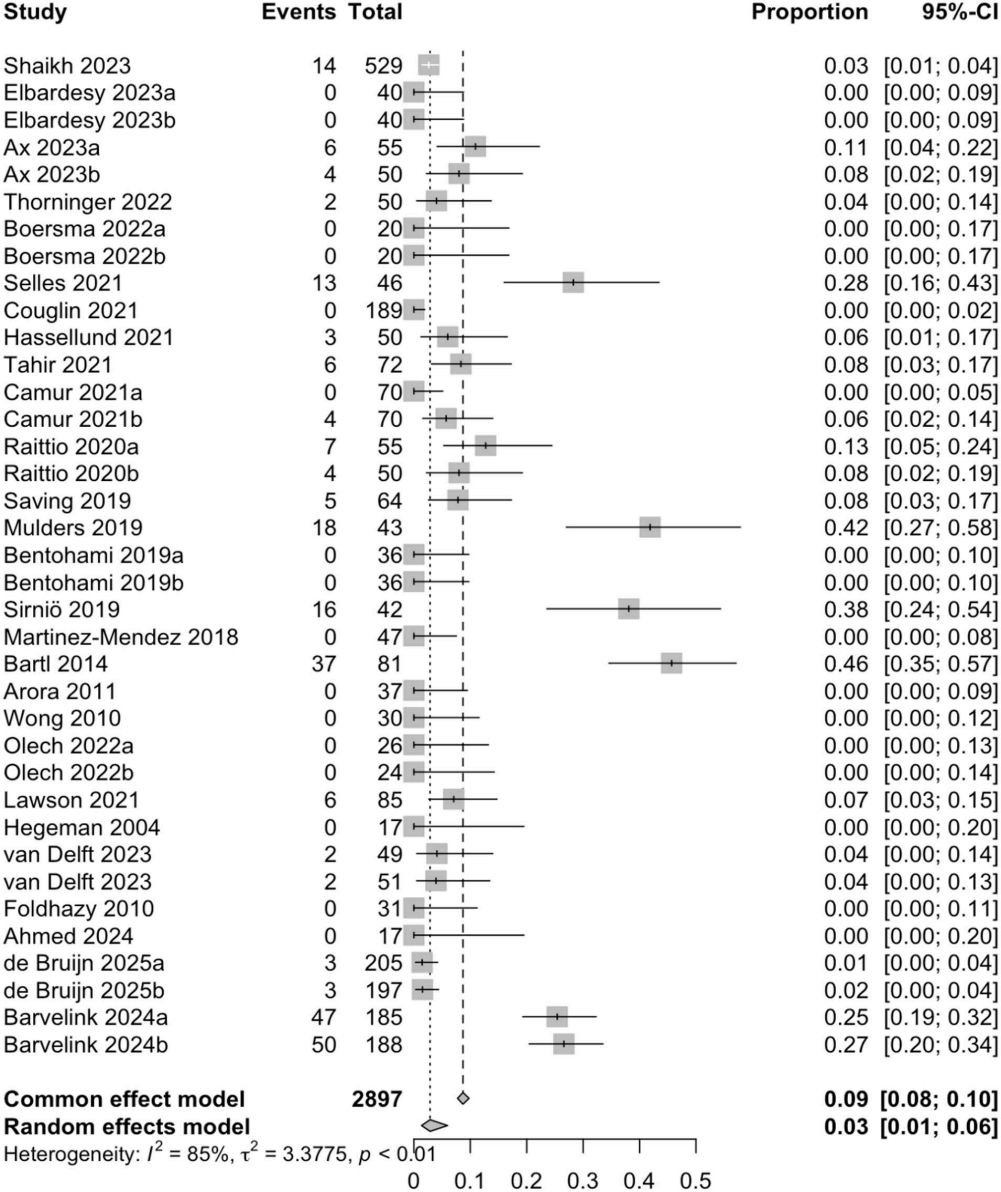
The pooled risk for the secondary surgery. *Note.* CI = confidence interval.

A total of 38 study arms with 3006 patients reported adverse events at the final follow-up. Of those, a total of 539 adverse events occurred, with a complication rate of 17.9%. The pooled risk of adverse events was 13.2% (3006 patients; CI = 8.8-19.3%; *I*^2^ = 89.3%). Loss of reduction was the most frequent complication, as it accounted for 54.5% (n = 294) of the complications. A total of 252 secondary surgeries were performed. The most common indication was loss of reduction, which accounted for 59.1% of the secondary surgeries, followed by corrective osteotomy (17.9%). The pooled risk of the secondary surgery was 2.9% (37 study arms; 2897 patients; CI = 1.4%-6.0%; *I*^2^ = 84.5%) (Figures 3 and 4).

### Health-Related Quality of Life

The most used measurement for HrQoL was SF-8, SF-12, or SF-36 (altogether 20 studies). At 3 months, the pooled mean of the combined outcome of SF-questionnaires was 43 (9 study arms; 870 patients; CI = 37.3-48.7; *I*^2^ = 98.9%). At 12 months, the pooled mean was 44.2 (7 study arms; 829 patients; CI = 39.3-49.1; *I*^2^ = 99.7%).

### Return to Work

One study reported^14^ outcomes, including the median time to return to work, which was 2.8 weeks.

## Discussion

This systematic review and meta-analysis reported the pooled PROM estimates at various follow-up time points in adult patients with nonoperatively treated DRFs. In addition, we examined the pooled pain, complication rates, and HrQoL at the specified time points. In the RCTs selected for the meta-analysis, the most frequently reported PROMs were PRWE, DASH, and qDASH.

The main finding of this study was that the pooled PRWE at 12 months was 10.8. This result suggests a relatively good functional outcome, as previous studies on operatively treated DRFs in RCT settings have consistently reported comparable or even higher PRWE scores at 12 months. At 12 months, Selles et al^30^ reported PRWE of 5 (44 patients, median age 62.0 years), and Lawson et al^23^ reported PRWE of 19.8 (81 patients, mean age 71.2 years). In addition, Mulders et al^38^ reported that the normative PRWE value in the general population has a mean of 7.7 (SD: 15.0), indicating similar wrist functionality in individuals without a DRF. Their study also found that PRWE scores increase significantly with age (mean 9.2 in patients aged 65 or more) and are higher in women.^38^ Comparing these findings to our results, the results suggest that the pooled PRWE was good compared with operatively treated DRFs, and that patients treated conservatively for a DRF achieve nearly normal wrist function within a year, particularly when considering the variability in PRWE scores within the general population.

Our meta-analysis shows that there is a significant improvement in the aforementioned PROMs between the 3-month and 12-month follow-ups. Earlier studies have reported that the minimal clinically important difference (MCID) is the smallest change perceived as important by the patient. The MCID is 11.5 for the PRWE, and 10 and 14 for the DASH and qDASH, respectively.^39^ Our study shows differences between the 3-month and 12-month follow-ups of 20.2 for the PRWE, 10.4 for the DASH, and 18.5 for the qDASH. There was no noticeable difference in the parameters measuring pain between the 3-month and 12-month measurement results. Similarly, there was no clear difference in the quality of life at these same time points.

Interestingly, we found that significant changes in PRWE, DASH, and qDASH occur between 3 and 12 months post-treatment. This suggests that full recovery of function may take at least 12 months. This information is crucial, as it allows health care providers to give patients accurate expectations regarding non-surgical treatment outcomes after DRF, which can be used to inform shared decision-making. As the number of studies was limited in this review, statistical analyses on the association between age and functional outcome were not performed. However, in most of the studies, the mean age of the patients was at least 65 years, indicating that our results might reflect the prognosis most accurately within that age group. In the future, our findings can serve as a valuable reference when discussing treatment options with patients.

In the study by McKay et al, where different treatment methods were separated, 27% of patients had physician-reported complications. The complication rate for non-surgically treated patients alone was 21%. In addition, they completed the 6-month follow-up questionnaire to evaluate patient-reported complications, with 21% of patients reporting complications.^40^ In our study, a total of 539 adverse events occurred. Nine study arms did not report complications, making calculating the exact complication rate impossible. The absence of reported complications could be because complication reporting was not part of the research plan or, alternatively, no complications occurred in those studies. Taking this into account, however, we can reliably estimate the complication rate in this study to range from 16% (539/3368) to 17.9% (539/3006), which is consistent with previous literature. A previously published meta-analysis reported a complication rate of 17% in patients with DRFs treated conservatively.^3^

### Strengths and Limitations

Although most DRFs are being treated nonoperatively with a cast, to the best of our knowledge, there has not been such an extensive review focused solely on patients with non-surgically treated DRFs. A key strength of our review is the inclusion of the latest RCT studies, which were not fully included in the previous systematic reviews. Some limitations of this study should be addressed. First, the RCTs did not report baseline values of PROMs, making it difficult to accurately assess the values classified as normal at 3 and 12 months. Individual variations in baseline values could affect how patients experience the fracture, further complicating the assessment. Second, although our analysis focused exclusively on non-surgically treated fractures, assuming low heterogeneity, some variability in fracture types was observed. This variability may influence the outcomes and should be considered when interpreting our findings.

## Conclusions

In conclusion, patients treated conservatively for a DRF achieve nearly normal wrist function within a year, particularly when considering the variability in PRWE scores within the general population.

In addition, we found significant improvement in PROMs, specifically PRWE, DASH, and qDASH, between the 3-month and 12-month follow-ups in adult patients with non-surgically treated DRFs. In addition, approximately 17% to 19% of patients with non-surgically treated DRFs experience adverse events following treatment. Further research is needed to identify the most effective treatment methods for different fracture types and patient groups, aiming to maximize patient benefit while minimizing complications.

## Supplemental Material

sj-docx-1-han-10.1177_15589447251415388 – Supplemental material for Non-Surgically Treated Distal Radius Fractures in the Adult Population: A Systematic Review and Meta-analysis Supplemental material, sj-docx-1-han-10.1177_15589447251415388 for Non-Surgically Treated Distal Radius Fractures in the Adult Population: A Systematic Review and Meta-analysis by Vili Palola, Rasmus Liukkonen, Matias Vaajala, Ilari Kuitunen, Antti P. Launonen and Ville M. Mattila in HAND

sj-docx-2-han-10.1177_15589447251415388 – Supplemental material for Non-Surgically Treated Distal Radius Fractures in the Adult Population: A Systematic Review and Meta-analysis Supplemental material, sj-docx-2-han-10.1177_15589447251415388 for Non-Surgically Treated Distal Radius Fractures in the Adult Population: A Systematic Review and Meta-analysis by Vili Palola, Rasmus Liukkonen, Matias Vaajala, Ilari Kuitunen, Antti P. Launonen and Ville M. Mattila in HAND
