# Supplementary material for: Non-Surgically Treated Distal Radius Fractures in the Adult Population: A Systematic Review and Meta-analysis
Source: Hand (N Y). 2026 Feb 1:15589447251415388. Online ahead of print. doi: 10.1177/15589447251415388 (PMC12864013; doi:10.1177/15589447251415388)
Supplement: sj-docx-2-han-10.1177_15589447251415388 – Supplemental material for Non-Surgically Treated Distal Radius Fractures in the Adult Population: A Systematic Review and Meta-analysis [file sj-docx-2-han-10.1177_15589447251415388.docx]

**Supplementary file 2. Risk of bias assessment template.**


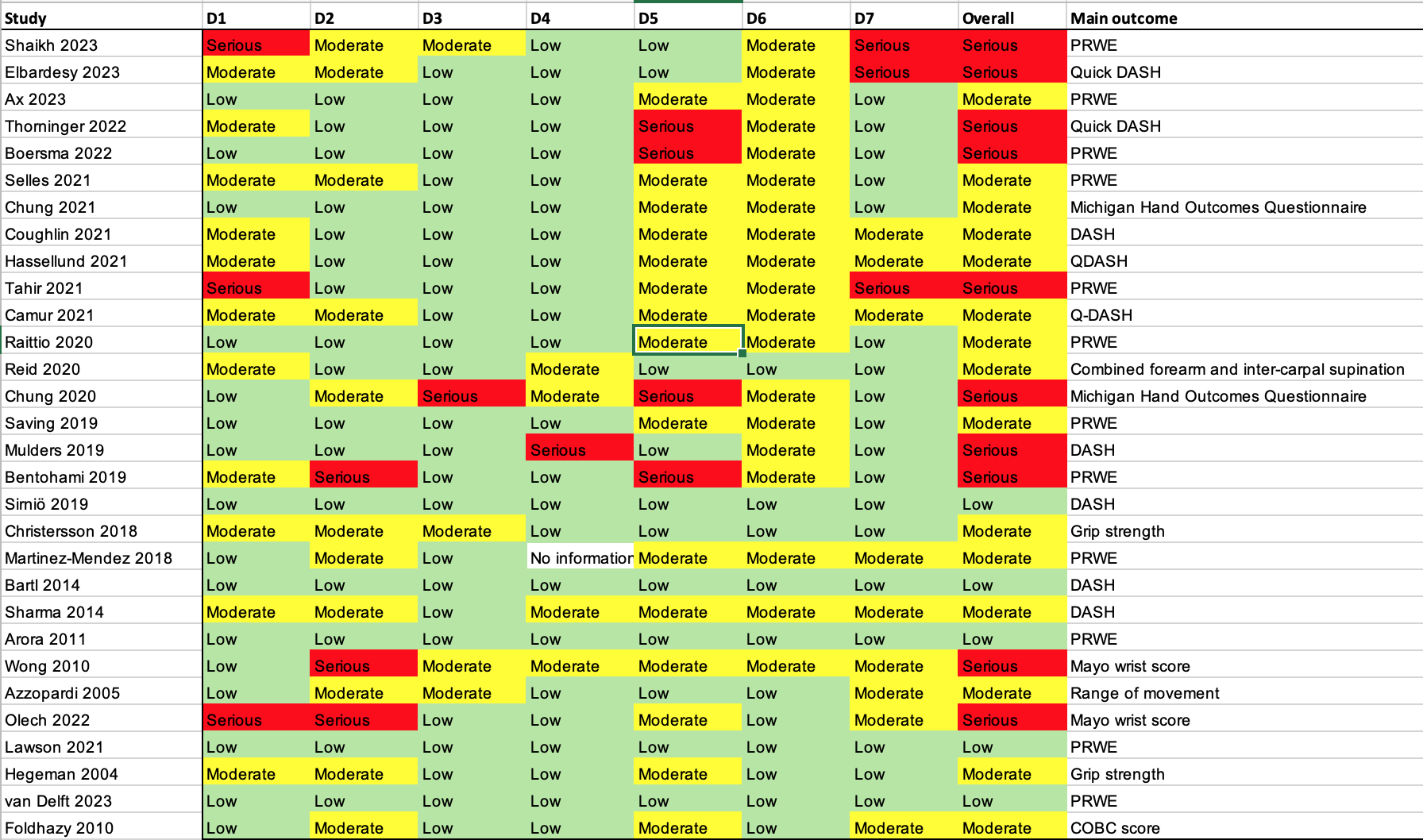


**Supplementary figure 1A-C. Funnel plot for the meta-analysis of pooled PRWE at the specified timepoints.**


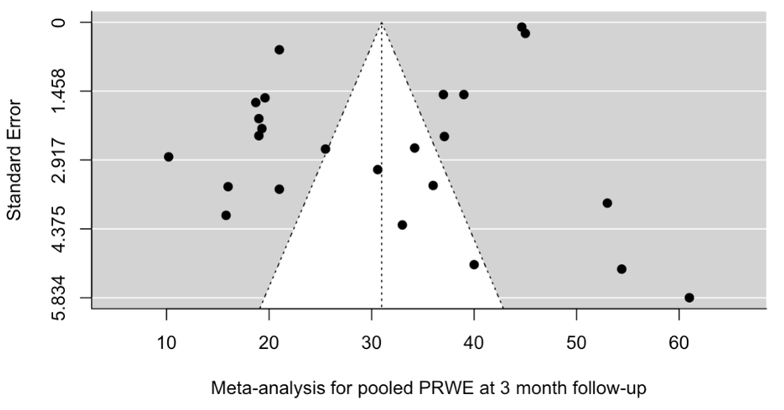


**Figure 1A. Funnel plot for the meta-analysis of pooled PRWE at 3-month follow-up.**


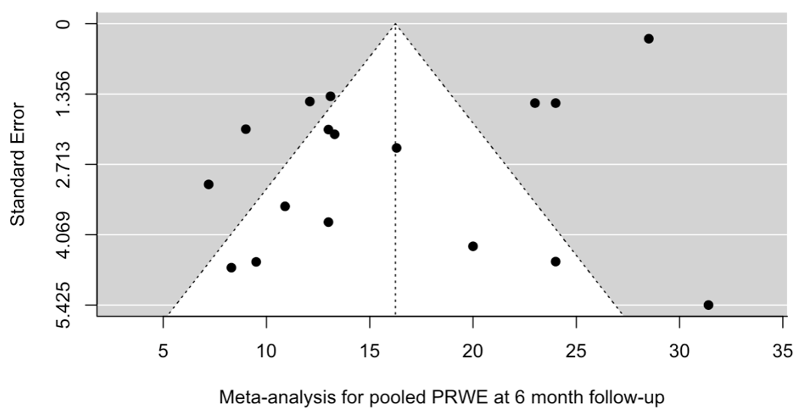


**Figure 1B.** **Funnel plot for the meta-analysis of pooled PRWE at 6-month follow-up.**


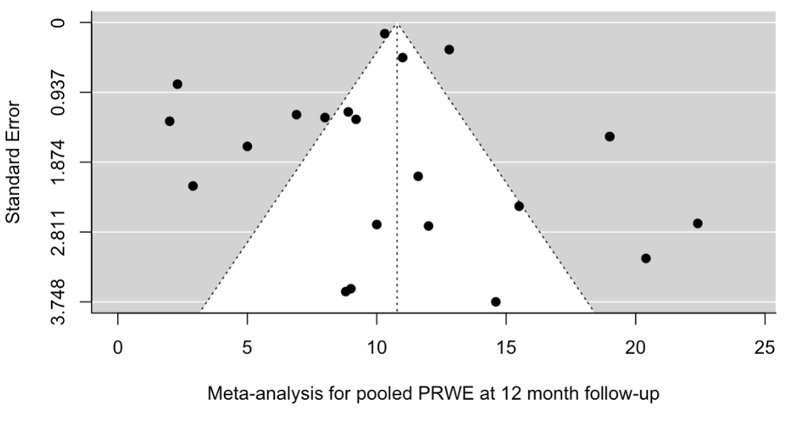


**Figure 1C.** **Funnel plot for the meta-analysis of pooled PRWE at 12-month follow-up.**
